# Photoelectrochemical Oxidation and Etching Methods Used in Fabrication of GaN-Based Metal-Oxide-Semiconductor High-Electron Mobility Transistors and Integrated Circuits: A Review

**DOI:** 10.3390/mi16101077

**Published:** 2025-09-23

**Authors:** Ching-Ting Lee, Hsin-Ying Lee

**Affiliations:** 1 Department of Photonics, National Cheng Kung University, Tainan 701, Taiwan; hylee@ee.ncku.edu.tw; 2 Institute of Microelectronics, Department of Electrical Engineering, National Cheng Kung University, Tainan 701, Taiwan; 3 Department of Electrical Engineering, Yuan Ze University, Taoyuan 320, Taiwan

**Keywords:** GaN-based devices, complementary metal-oxide-semiconductor inverters, metal-oxide-semiconductor high-electron mobility transistors, photoelectrochemical etching method, photoelectrochemical oxidation method

## Abstract

The photoelectrochemical oxidation method was utilized to directly grow a gate oxide layer and simultaneously create gate-recessed regions for fabricating GaN-based depletion-mode metal-oxide-semiconductor high-electron mobility transistors (D-mode MOSHEMTs). The LiNbO_3_ gate ferroelectric layer and stacked gate oxide layers of LiNbO_3_/HfO_2_/Al_2_O_3_ were respectively deposited on the created gate-recessed regions using the photoelectrochemical etching method to fabricate the GaN-based enhancement mode MOSHEMTs (E-mode MOSHEMTs). GaN-based complementary integrated circuits were realized by monolithically integrating the D-mode MOSHEMTs and the E-mode MOSHEMTs. The performances of the inverter circuit manufactured using the integrated GaN-based complementary MOSHEMTs were measured and analyzed.

## 1. Introduction

Gallium nitride (GaN)-based semiconductors are direct wide-bandgap materials with an energy bandgap of approximately 3.4 eV. Compared with conventional silicon (Si)-, gallium arsenide (GaAs)-, and indium phosphide (InP)-based semiconductor materials, GaN exhibits an exceptionally high critical electric field (~3.3 MV/cm), enabling GaN-based devices to operate at high temperature and high voltage without breakdown [1]. Moreover, GaN-based semiconductors have a high electron saturation velocity (~2.5 × 10^7^ cm/s), enhancing high-frequency performance. In addition, the room-temperature electron mobility in a GaN-based two-dimensional electron gas (2DEG) heterostructure is around 1500–2000 cm^2^/V·s, allowing for reduced on-resistance and increased output power in applications [2]. Accordingly, the wide bandgap, high electron mobility, high saturation velocity, and large breakdown field of GaN-based semiconductors underpin their enormous promising applications for low-noise, high-power, and high-frequency systems [3,4,5,6]. Owing to these advantageous properties, GaN-based devices have been successfully applied in 5G mobile-communication base stations, smartphones and tablets, electric vehicle chargers, data-center power supplies, radar transmit/receive modules, photovoltaic microinverters, and medical-imaging equipment.

**Figure 1:**
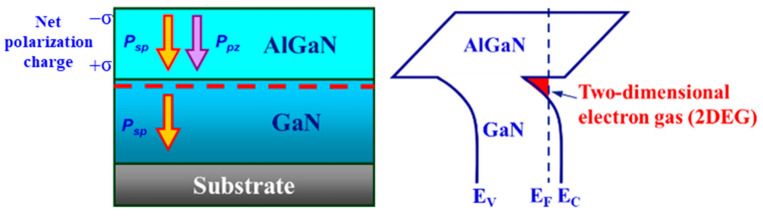
Generation of two-dimensional electron gas between AlGaN/GaN heterojunction.

GaN-based high-electron mobility transistors (HEMTs) typically employ an AlGaN/GaN heterostructure to form a conduction channel. Spontaneous polarization and piezoelectric polarization exist in the interface between the AlGaN and GaN layers, which induce a high-density two-dimensional electron gas, as shown in Figure 1, yielding high room-temperature electron mobility. This high-density, high-mobility 2DEG provides an excellent conduction channel, resulting in GaN-based HEMTs that deliver large drain currents and low on-resistance. Their high-frequency performances are also outstanding, with maximum oscillation frequency (f_max_) reaching several hundred gigahertz [7]. In summary, the 2DEG with high carrier concentration and high mobility formed by the AlGaN/GaN heterointerface is the core of the excellent performance of GaN-based HEMTs, enabling them to have low-noise, high-power, high-frequency, and high-temperature working capabilities and functions.

Although GaN-based metal-semiconductor HEMTs (MESHEMTs) with a Schottky-gate structure have garnered significant attention and are widely deployed [8,9], their performance is fundamentally limited by the low Schottky barrier height and interface defects inherent to the metal–semiconductor junction, which give rise to high gate leakage currents, current collapse, and reduced breakdown voltages [10,11]. To meet the stringent requirements of low-noise, high-frequency, and high-power applications, metal-oxide-semiconductor HEMTs (MOSHEMTs) have been developed by inserting an insulating oxide layer between the AlGaN layer and the gate metal. For the gate dielectric in MOSHEMTs, the film must exhibit a high resistivity, a large breakdown field, excellent chemical stability, and low interface-state density. Among candidate dielectrics, wide-bandgap metal oxide semiconductors and stacked dielectric layers, such as ZnO, Ga_2_O_3_, Al_2_O_3_, SiO_2_, Si_3_N_4_, and Al_2_O_3_/ZrO_2_, et al., have attracted particular interest [12,13,14,15]. However, these gate oxide layers deposited by conventional methods, such as sputtering, pulsed-laser deposition, atomic-layer deposition, or chemical-vapor deposition, typically contain inevitable contamination and interface states. It is seen that the direct growth of silicon dioxide on silicon by wet and thermal oxidation methods, drastically reducing contamination and interface state density, is an important factor and key technology in the successful manufacture of silicon devices and integrated circuits. Analogously, a method for direct oxide growth on GaN-based semiconductors would similarly suppress contamination and interface defects, thereby improving device characteristics. To achieve this goal, we developed a photoelectrochemical (PEC) oxide method to directly grow high-quality and low interface state density gate oxide films on the surfaces of GaN-based semiconductors and simultaneously created gate-recessed regions. Moreover, to increase breakdown voltage and output power, the electric-field distribution in the channel must be smoothed to avoid localized field spikes. A common solution was to form a gate-recessed region beneath the gate electrode of the MOS devices. However, the conventional plasma etching method used to create these gate-recessed regions damaged the GaN-based material, and subsequent dielectric deposition might introduce additional interface defects. The PEC etching method was developed and used to create gate-recession regions for fabricating GaN-based MOSHEMTs.

## 2. Photoelectrochemical Oxidation and Etching Methods

**Figure 2:**
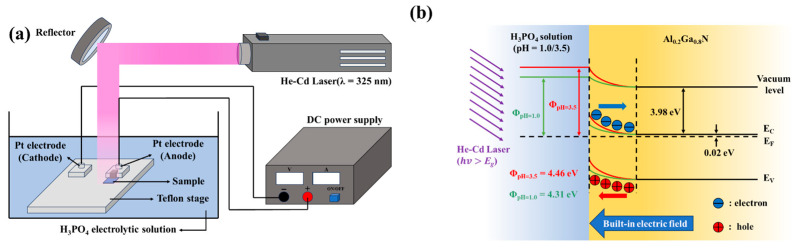
(**a**) Schematic diagram of photoelectrochemical oxidation/etching system and (**b**) energy diagram between AlGaN layer and H_3_PO_4_ electrolytic solution with a pH value of 1.0 and 3.5.

Figure 2a shows the photoelectrochemical oxidation/etching system. The GaN-based samples were immersed in a He-Cd laser (wavelength = 325 nm) illuminated phosphoric-acid (H_3_PO_4_) electrolytic solution with various pH values. The work function W_E_ of the H_3_PO_4_ solution and work function W_S_ of the AlGaN layer were respectively expressed as [16]

W_E_ (eV) = 4.25 + 0.059 × pH value (1)

W_S_ (eV) = 3.98 + (E_C_ − E_F_) (2)

where 3.98, E_C_, and E_F_ were the electron affinity, conduction band, and Fermi level of the AlGaN layer, respectively. Figure 2b shows the bandgap energy diagram between the Al_0.2_Ga_0.8_N layer (electron concentration = 1.2 × 10^18^ cm^−3^) and the H_3_PO_4_ solution with a pH value of 1.0 and 3.5, respectively. Due to the bent bandgap energy at the interface, a built-in electric field was also induced. Because the photon energy of the He-Cd laser was larger than the bandgap energy of the AlGaN layer, electron-hole pairs were generated in the surface of the AlGaN layer. The generated holes and electrons were driven to the surface and the inside of the GaN-based layer by the induced built-in electric field and the applied direct current (DC) voltage, respectively. The holes (h^+^) interacted with the GaN layer and the AlGaN layer and oxidized the GaN layer and the AlGaN layer as described by the following formula:

(3) $$2\mathrm{GaN}+6h^{+}+3H_{2}O\;⇆\;{\mathrm{Ga}}_{2}O_{3}+N_{2}+6H^{+}$$

(4) $$2\mathrm{AlGaN}+12h^{+}+6H_{2}O⇄{\mathrm{Al}}_{2}O_{3}+{\mathrm{Ga}}_{2}O_{3}+N_{2}+12H^{+}$$

It was found that the Ga_2_O_3_ and the mixed Ga_2_O_3_ and Al_2_O_3_ oxide materials were formed on the GaN surface and the AlGaN surface, respectively. While the oxide materials were growing, they were also simultaneously etched by the H_3_PO_4_ electrolytic solution. In general, the growth and etching speeds of the oxide layer depended on the pH value of the H_3_PO_4_ solution. If the oxidation rate was larger than the etching rate, the gate oxide layer could be directly grown. Otherwise, the AlGaN layer could be etched to create gate-recessed regions. Not only could n-type GaN-based semiconductors be oxidized, but p-type GaN-based semiconductors could also be oxidized by applying a bias-assisted PEC oxidation method [17].

**Figure 3:**
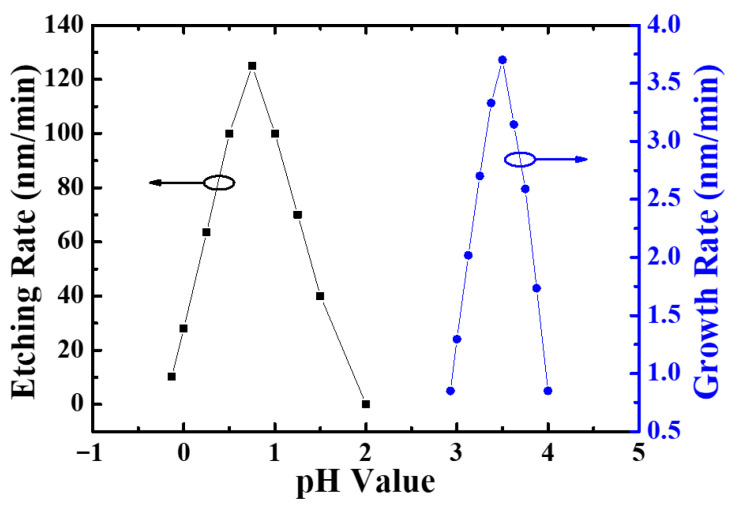
Oxidation rate and etching rate of GaN layers as a function of pH value of H_3_PO_4_ solution.

**Figure 4:**
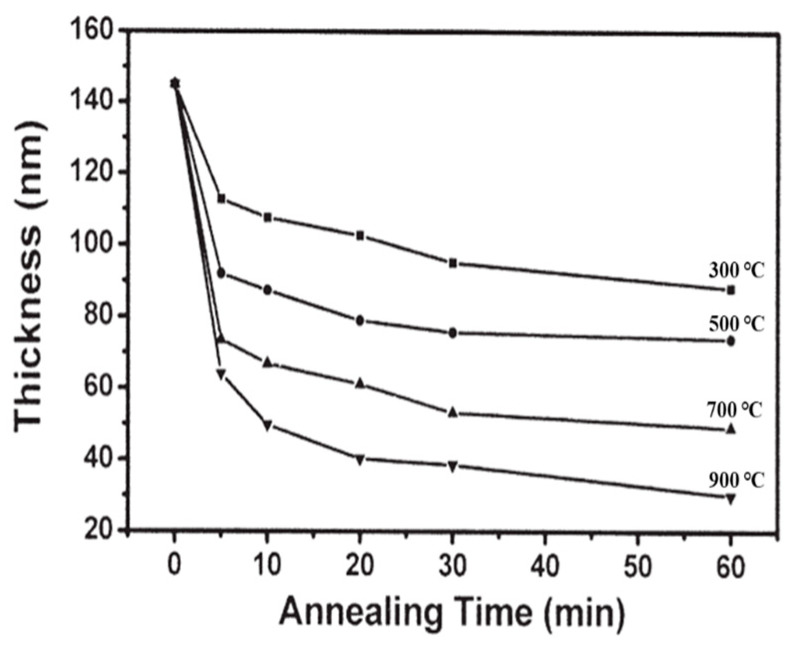
Oxide layer thickness as a function of annealing temperature for various annealing times [19].

**Figure 5:**
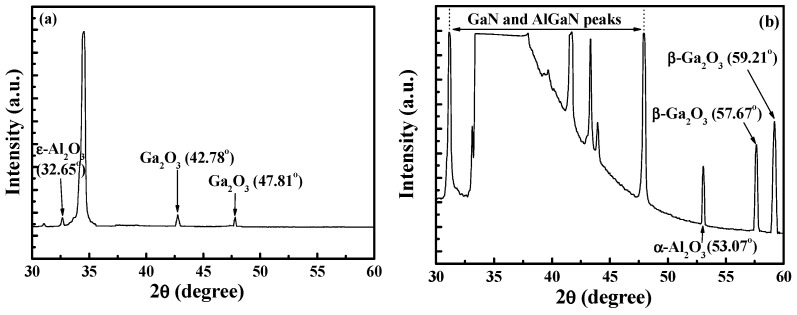
X-ray diffraction patterns of photoelectrochemical oxide layer (**a**) without and (**b**) with annealing in O_2_ ambient at 700 °C for 2 h.

Figure 3 shows the growth rate and etching rate of GaN layers as a function of the pH value of the H_3_PO_4_ electrolytic solution [18,19]. It could be seen that the gate oxide layer could be directly grown when a pH value of 3.5 was used, while the GaN-based layer could be directly etched to form gate-recessed regions using a pH value of 1.0. Similar to the experimental results of growing a silicon dioxide layer on a silicon layer using a wet method or a thermal method, the thickness of the grown silicon dioxide layer was larger than the consumed thickness of the silicon layer. In the experimental PEC oxidation results, the thickness of the grown oxide layer was larger than the consumed thickness of the GaN-based layer. If the thickness of the oxide layer was t_ox_, the consumed thickness of the GaN-based layer was approximately 0.33 t_ox_ [19]. Since the oxide layer directly grown by the PEC oxidation method was too soft and revealed poor crystal quality, it was etched by the developer during the photolithography process. Therefore, it could not be successfully used to manufacture GaN-based MOSHEMTs. To improve the quality of the oxide layers for manufacturing devices, the directly grown oxide layers were annealed in an oxygen environment at various conditions. Figure 4 shows the dependence of the thickness of the grown oxide layer on the annealing temperatures for various times [19]. Under the same annealing time, it was found that the thickness of the oxide layer decreased with an increase in annealing temperature. Similarly, under the same annealing temperature, the thickness of the oxide layer rapidly decreased and then gradually decreased with annealing time. Furthermore, the refractive index of the annealed oxide layer would be slightly increased with an increase in annealing time and annealing temperature. From these experimental results, it could be deduced that the residual materials would be sublimated, and the density of the grown oxide layer became denser during the annealing process. Figure 5a,b show the X-ray diffraction patterns of the mixed Ga_2_O_3_ and Al_2_O_3_ oxide layers without and with annealing at 700 °C for 2 h, respectively. Using the annealing process, Ga_2_O_3_ was transferred to β-Ga_2_O_3_ and ε-Al_2_O_3_ was transferred to α-Al_2_O_3_. It was found that the high-quality mixed β-Ga_2_O_3_ and α-Al_2_O_3_ crystalline oxide layer was obtained [20]. The resulting stable oxide layer could prevent damage from the developer, alkaline chemical solution, and acidic chemical solution. Consequently, the annealed oxide layers grown by the PEC oxidation method could be used for fabricating GaN-based MOSHEMTs. Using the laser photoassisted capacitance-voltage measurements under a Xe-lamp (wavelength = 325 nm) illumination [21], the interface state density between the PEC grown oxide layer and the GaN-based layer of 5.1 × 10^11^ eV^−1^cm^−2^ was obtained, demonstrating that the PEC oxidation method could directly produce gate oxide layer on GaN-based layer with high quality and low interface state density.

## 3. Photoelectrochemical Fabrication Function in GaN-Based D-Mode MOSHEMTs

The epitaxial layers of the GaN-based D-mode MOSHEMTs were grown on C-plane sapphire substrates using an ammonia molecular-beam epitaxy system. The epitaxial layers were designed from bottom to top, including an AlN nuclear layer (20 nm), a carbon-doped GaN buffer layer (1.7 μm), an undoped GaN layer (0.5 μm), and an undoped Al_0.2_Ga_0.8_N layer (35 nm). Under the Hall measurement at room temperature, the sheet electron density and electron mobility on the 2DEG channel were 8.89 × 10^12^ cm^−2^ and 1460 cm^2^/V·s, respectively.

**Figure 6:**
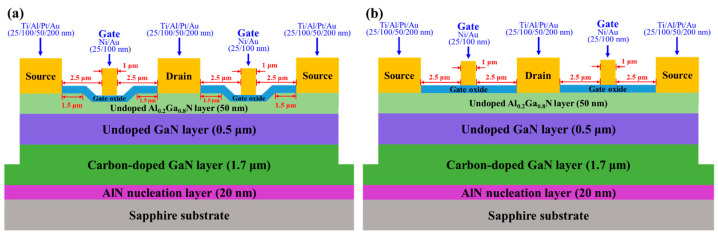
Epitaxial layers and schematic configuration of (**a**) gate-recessed and (**b**) planar GaN-based D-mode MOSHEMTs.

Using a 300 nm-thick Ni mask, the mesa isolation regions (215 × 375 μm^2^) were created by etching the sample until the carbon-doped GaN layer using a BCl_3_ etchant in a reactive-ion-etching system. To completely remove the native oxide that resided on the surface of the undoped Al_0.2_Ga_0.8_N layer, surface treatment was carried out by dipping the samples into (NH_4_)_2_S_x_ (S = 0.6%) solution at 60 °C for 20 min [22,23]. Since the following gate oxide layer grown by the PEC oxidation method should be annealed at a higher temperature, the long-term stable stacked Ti/Al/Pt/Au (25/100/50/200 nm) multiple metals were deposited as the source electrode and the drain electrode using an electron-beam evaporator [24]. They were annealed in an N_2_ ambient rapid-thermal-annealing system at 850 °C for 2 min to form ohmic performances. By illuminating a He-Cd laser (power density = 10.0 mW/cm^2^ and wavelength = 325 nm) onto the surface of the samples in a H_3_PO_4_ electrolytic solution with a pH value of 1.0, the PEC etching method was utilized to create 8 nm-deep gate-recessed regions on the windows opened by a standard photolithography method. Similarly, the H_3_PO_4_ electrolytic solution with a pH value of 3.5 was employed to directly grow the gate oxide layer using the PEC oxidation method. The grown oxide layers were then annealed in an oxygen ambient furnace at 700 °C for 2 h to obtain a stable β-Ga_2_O_3_ and α-Al_2_O_3_ oxide layer. The annealed oxide layer acted not only as the gate oxide layer but also as a surface passivation layer of the Al_0.2_Ga_0.8_N layer. Using an electron-beam evaporator and a liftoff process, two-finger Ni/Au (20/100 nm) gate metals with 1-μm length and 50-μm width were formed. The epitaxial layers and the schematic configuration of the GaN-based D-mode MOSHEMTs with gate-recessed structure were illustrated in Figure 6a. Using the same epitaxial layers and the same fabrication processes, the planar gate GaN-based D-mode MOSHEMTs without a gate-recessed structure were also illustrated in Figure 6b.

**Figure 7:**
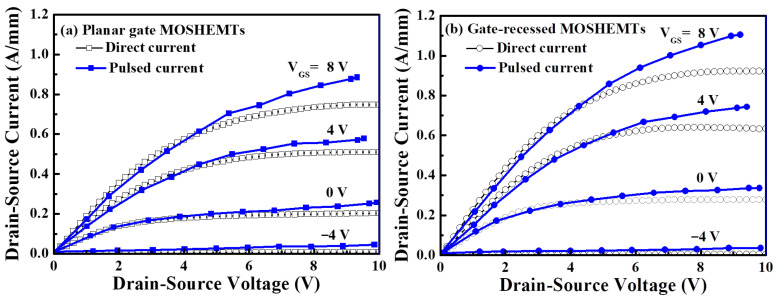
Direct current and pulsed I_DS_-V_DS_ characteristics of GaN-based (**a**) planar gate and (**b**) gate-recessed MOSHEMTs.

**Figure 8:**
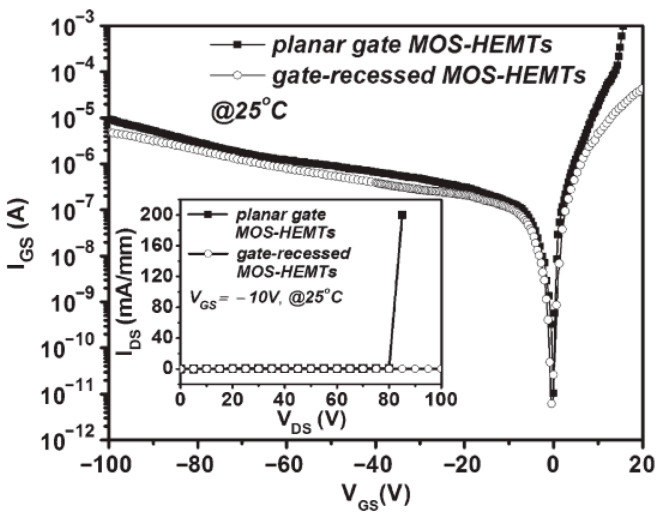
Gate-source leakage current as a function of gate-source voltage of GaN-based planar gate and gate-recessed MOSHEMTs [26].

Using the measurement of Agilent 4156C semiconductor parameter analyzer, Figure 7a,b show the direct current (DC) drain-source current (I_DS_)-drain-source voltage (V_DS_) characteristics and pulsed I_DS_-V_DS_ characteristics of the planar gate and gate-recessed GaN-based D-mode MOSHEMTs, respectively. To measure the pulsed I_DS_-V_DS_ characteristics of the devices, an Accent dynamic I (V) analyzer (DIVA) model D225 with a pulse width of 100 ns and 1 ms separation between each pulse signal was employed. The quiescent bias point of the measurement was V_GS_ = 0 V and V_GS_ = V_pinch-off_, respectively, where V_pinch-off_ was the pinch-off voltage [25]. At the V_GS_ of 0 V, the saturation drain-source currents and threshold voltages of the planar gate and gate-recessed devices were 509 and 642 mA/mm, and −8.5 and −8.0 V, respectively [26]. Since the thickness of the AlGaN under the gate metal of the gate-recessed device was smaller than that of the planar gate device, the absolute value of the threshold voltage was correspondingly smaller. As shown in Figure 7a,b, the fact that the pulsed I_DS_ value was larger than the direct current I_DS_ value for the devices operated at the saturation region was attributed to the self-heating effect [27]. In addition, the planar gate and gate-recessed GaN-based MOSHEMTs exhibited similar pulsed output characteristics. Compared with the I_DS_ value, it was found that the gate-recessed devices not only did not exhibit a significant transient drain-source current reduction in the linear region, but also no gate lag phenomenon or current collapse phenomenon was observed. In general, the gate lag was induced by the electron traps caused by the defects and surface states [28]. The experimental results verified that the PEC process produced fewer defects and surface states during the fabrication of devices. Figure 8 illustrates the gate-source leakage current as a function of the gate-source voltage of the planar gate and gate-recessed GaN-based MOSHEMTs. Under the reverse-biased gate-source voltage of −100 V, the gate-source leakage currents of the planar gate and gate-recessed devices were 9.32 μA and 4.63 μA, respectively. This experimental result not only verified that the PEC etching method could be used to create gate-recessed regions, but also that the PEC oxidation method could grow a gate oxide layer with properties of a high breakdown electric field and surface passivation layers.

**Figure 9:**
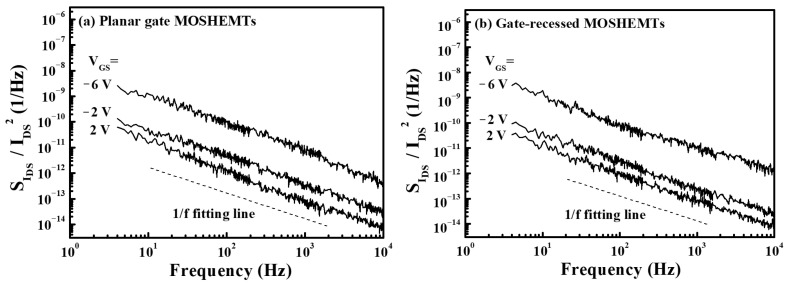
Normalized low frequency noise power density of GaN-based (**a**) planar gate and (**b**) gate-recessed MOSHEMTs.

An HP 4145B semiconductor parameter analyzer, an HP 35670A dynamic signal analyzer, and a BTA noise analyzer were used to measure the low frequency noise performances at room temperature. By defining that $S_{I_{DS}}$ was the noise spectral density as a function of drain-source current (I_DS_), Figure 9a,b illustrate the normalized noise power density ($S_{I_{DS}}/{I_{DS}}^{2}$) at a low frequency of the planar gate and gate-recessed GaN-based MOSHEMs operating at a drain-source voltage (V_DS_) of 1 V and various gate-source voltages. It could be found that the normalized noise power density had a relatively well-defined tendency of 1/f. This result could be ascribed to the dominant noise originating from the flicker noise. As shown in Figure 9a,b, both devices had similar normalized noise power density values. Since the low frequency noise was significantly affected by the surface damage and the induced defects [29,30,31], the experimental result of the similar normalized low frequency noise power density clearly verified that the PEC etching method and the PEC oxidation method did not cause surface damage and induce defects during the fabrication of the GaN-based MOSHEMTs.

## 4. Photoelectrochemical Fabrication Function in GaN-Based E-Mode MOSHEMTs

In the practical design and applications of integrated circuits, in addition to requiring GaN-based D-mode MOSHEMTs, the circuit architecture integrated with GaN-based E-mode MOSHEMTs can possess inherent advantages, including circuit design simplicity, circuit operation security, and low power consumption [32]. The depletion of 2DEG at the AlGaN/GaN heterostructure interface was essential in fabricating GaN-based E-mode HEMTs. The GaN-based E-mode HEMTs would be qualified with a larger threshold voltage and a larger electrical transport for the undamaged 2DEG channel at the AlGaN/GaN interface. In the past year, GaN-based E-mode HEMTs were successfully manufactured using several technologies, including fluorine plasma treatment [33,34], p-type GaN capping layer [35,36], gate-recessed structure [37,38], ferroelectric gate oxide layer [39,40], and oxide charge engineering [41,42]. However, the resulting E-mode devices fabricated using each technology exhibited different advantages and disadvantages. In addition, each technology had its critical difficulty to obtain the best characteristics. Using the gate-recessed structure, the depth of the gate-recessed regions created using etching technology must be carefully controlled to avoid the destruction of the 2DEG channel and the electron mobility caused by the over-etching process. On the other hand, if the gate-recessed regions were not dug deep enough, the remaining 2DEG would not enable the manufactured devices to obtain optimal enhancement mode characteristics. A combination technique of partial gate recess and fluorine implantation or ZrO_x_ charge trapping layer was reported previously [43,44]. Although several ferroelectric materials, such as Pb(ZrTi)O_3_ (PZT), BaTiO_3_ (BTO), HfZrO_2_, and LiNbO_3_, were used for fabricating GaN-based E-mode HEMTs [45,46,47], they should have strong ferroelectric polarization properties to achieve high enhancement mode characteristics. Furthermore, by regulating the polarization state of the ferroelectric gate layer, the 2DEG density and the corresponding threshold voltage could be continuously modulated. Therefore, the operating mode of the GaN-based MOSHEMTs could be switched between E-mode and D-mode for use in high-speed logic systems. Among the ferroelectric materials, crystalline lithium niobate (LiNbO_3_) material was one of the promising candidates owing to its wide bandgap of 3.9 eV and high spontaneous polarization (P_s_ = 70~80 μc/cm^2^) in the C^+^ domain [48], in which the polarization direction was opposite to that of GaN-based polarization to deplete 2DEG. Moreover, low interface state density between the LiNbO_3_ film and the GaN-based semiconductors was demonstrated previously [49,50]. To minimize the risk of 2DEG channel damage and to relax the tolerance of the manufacturing process of the GaN-based E-mode MOSHEMTs, the technologies of using the PEC etching method to create gate-recessed regions and the pulsed krypton fluoride (KrF) laser to deposit ferroelectric LiNbO_3_ film to work as the gate oxide layer were demonstrated previously [51].

**Figure 10:**
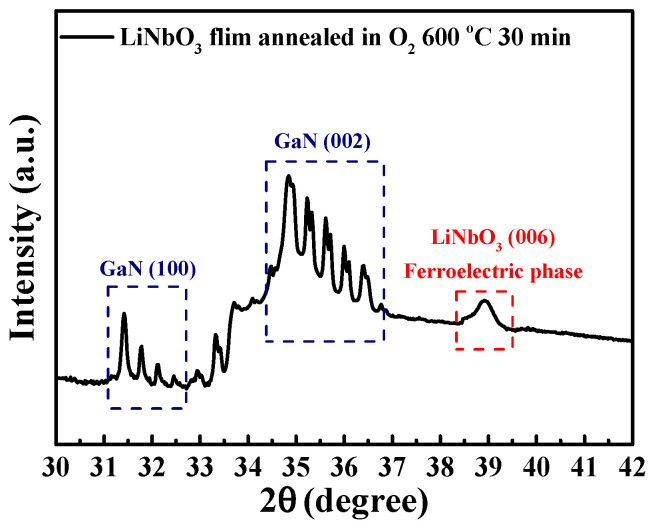
θ-2θ X-ray diffraction pattern of LiNbO_3_ film annealed in oxygen ambience at 600 °C for 30 min.

**Figure 11:**
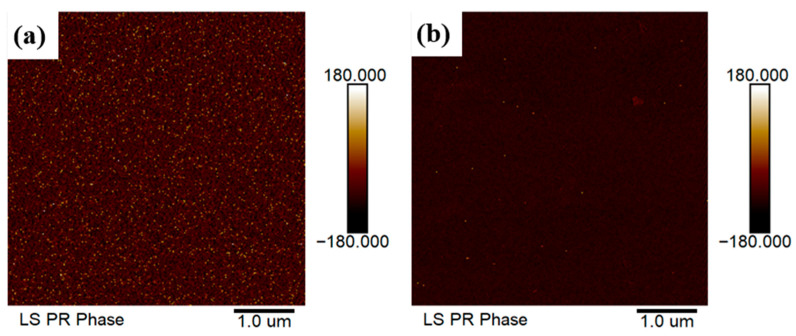
Vertical piezoelectric force microscopy image of LiNbO_3_ films (**a**) without annealing and (**b**) with annealing in oxygen atmosphere at 600 °C for 30 min.

A pulsed KrF laser deposition system was utilized to deposit 150 nm-thick LiNbO_3_ films onto the surfaces of the samples used for fabricating those above-mentioned GaN-based D-mode MOSHEMTs. To obtain spontaneous polarization perpendicular to the sample and opposite to the polarization direction of the GaN-based epitaxial layers, the strong (006) crystalline ferroelectric phase of the LiNbO_3_ films was obtained by annealing them in an oxygen atmosphere at 600 °C for 30 min [51]. The resulting X-ray diffraction pattern is shown in Figure 10. It was clearly found that the (006) crystalline ferroelectric phase resulted from the annealed LiNbO_3_ films. Figure 11a,b show the vertical piezoelectric force microscopy images of the LiNbO_3_ films without and with annealing in an oxygen atmosphere at 600 °C for 30 min. Using the vertical scanning model, the dark and bright colors shown in Figure 11a,b revealed the polarization in the C^+^ domain and the C^−^ domain, respectively. It was found that the annealed LiNbO_3_ film revealed almost C^+^ polarization orientation.

**Figure 12:**
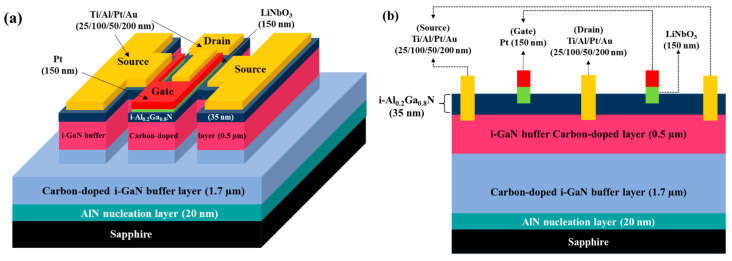
(**a**) Three-dimensional and (**b**) cross-sectional schematic configurations of LiNbO_3_/AlGaN/GaN E-mode MOSHEMT.

**Figure 13:**
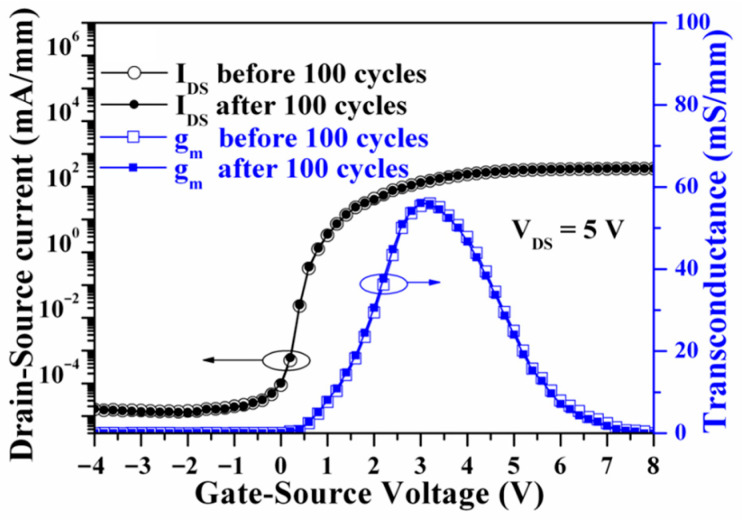
Transfer characteristics of LiNbO_3_/AlGaN/GaN E-mode MOSHEMTs before and after repeated operation for 100 cycles [51].

**Figure 14:**
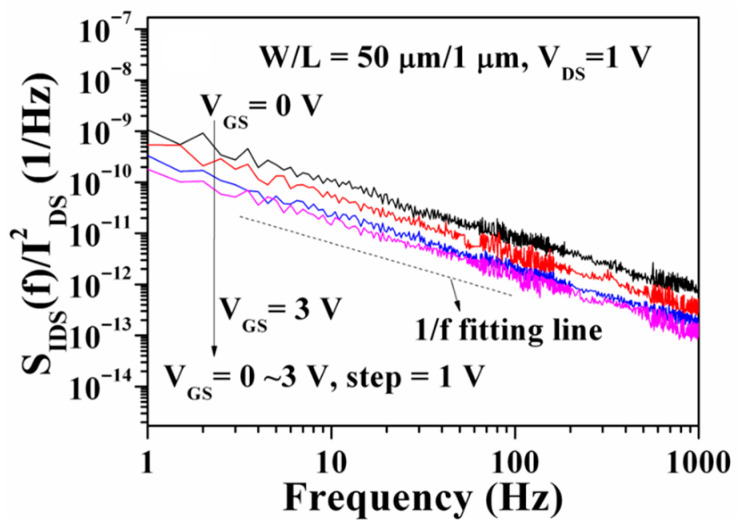
Normalized noise power density as a function of frequency of LiNbO_3_/AlGaN/GaN E-mode MOSHEMTs [51].

The epitaxial layers used for fabricating GaN-based E-mode MOSHEMTs were the same as those used for fabricating the above-mentioned D-mode devices. To investigate the effect of the remaining AlGaN thickness underneath the gate-recessed regions on the characteristics of devices, various deep gate-recessed regions were created using the PEC etching method. Because of the suppression of the polarization-induced charge effect, the sheet electron density and the electron mobility in the 2DEG channel would gradually reduce with a decrease in the AlGaN thickness [52]. When the remaining AlGaN thickness was only 5 nm, no electron concentration could be measured using the Hall measurement. This phenomenon was attributed to the suppression of polarization and the destruction of the 2DEG channel. Except for using a pulsed KrF laser deposition system to deposit LiNbO_3_ films on the PEC-etched gate-recessed regions and then annealing them in an atmosphere at 600 °C for 30 min, the rest of the manufacturing process of D-mode devices and E-mode devices was the same. Figure 12a,b show the three-dimensional and cross-sectional schematic configuration of the LiNbO_3_/AlGaN/GaN E-mode MOSHEMTs, respectively. When the I_DS_-V_GS_ characteristics of the devices with various remaining AlGaN thicknesses were measured under V_DS_ of 5 V, it was found that the threshold voltage moved toward the positive voltage direction as the remaining thickness of the AlGaN layer decreased. When the remaining thickness was 15 nm, the associated threshold voltage was 0.40 V. The positive threshold voltage verified that the GaN-based E-mode MOSHEMTs could be obtained using the partially gate-recessed structure and the LiNbO_3_ ferroelectric gate oxide layer. To study the reliability of the 150 nm-thick LiNbO_3_ ferroelectric gate oxide layer in the GaN-based E-mode MOSHEMTs, the devices were repeatedly operated for 100 cycles under the V_DS_ of 5 V and various V_GS_ voltages. Figure 13 shows the associated transfer characteristics before and after repeated operation for 100 cycles. After the devices were repeatedly operated for 100 cycles, the threshold voltage, subthreshold swing, and maximum transconductance were approximately 0.39 V, 427.3 mV/decade, and 56.1 mS/mm, which were similar to the original values of 0.40 V, 424.7 mV/decade, and 56.0 mS/mm, respectively. These experimental results verified the high reliability performances of the LiNbO_3_ ferroelectric gate oxide layer. Figure 14 shows the low frequency noise performance of the normalized noise power density ($S_{I_{DS}}(f)/{I_{DS}}^{2}$) a function of frequency (f) of the devices operated at V_DS_ of 1 V. The normalized power density of the devices operated at a frequency of 10 Hz, V_GS_ of 0 V, and V_DS_ of 1 V was 8.1 × 10^−11^ Hz^−1^. It was found that the normalized noise power density decreased with frequency and gate-source voltage. As shown in Figure 14, because the normalized noise power density revealed a well-defined tendency of 1/f, the dominant noise was deduced to originate from the flicker noise.

**Figure 15:**
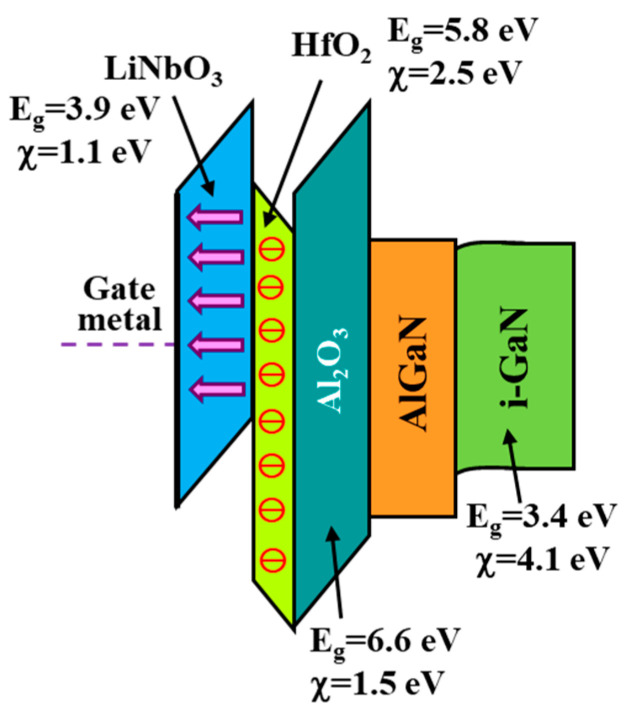
Energy band diagram of LiNbO_3_/HfO_2_/Al_2_O_3_ on AlGaN/GaN.

**Figure 16:**
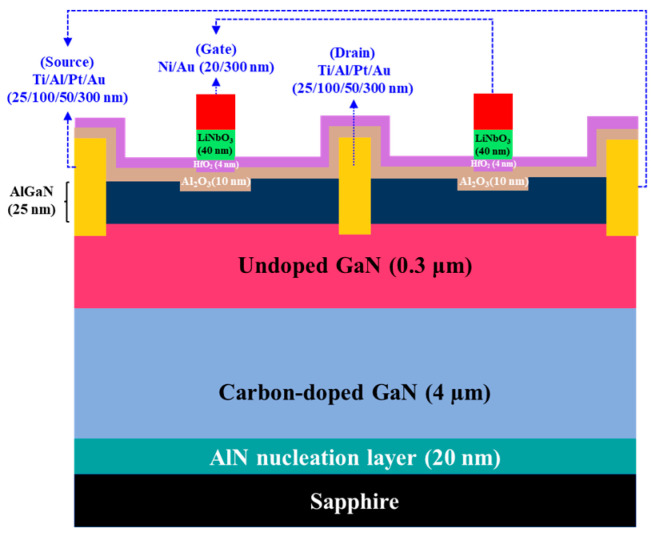
Cross-sectional schematic configuration of GaN-based E-mode MOSHEMTs with ferroelectric charge-trapping gate stacked of LiNbO_3_/HfO_2_/Al_2_O_3_ layers.

Even though GaN-based E-mode HEMTs could be fabricated using a single ferroelectric gate layer, a ferroelectric charge-trapping gate stack was developed and demonstrated in GaN-based E-mode MOSHEMTs to enhance enhancement performances [53,54,55,56,57,58]. The conventional ferroelectric charge-trapping gate stack generally included a tunneling oxide layer, a charge-trapping layer, and a blocking oxide layer. Although the SiO_2_ layer was used as the tunneling oxide layer [59], it was demonstrated that the low interface state density and low leakage current were obtained by using an atomic layer deposition (ALD) system to deposit an Al_2_O_3_ layer onto the GaN layer [60]. Consequently, the ALD-deposited Al_2_O_3_ layer can be used as the tunneling oxide layer. Furthermore, wide bandgap HfO_2_-based ferroelectric materials have remanent polarization and a high breakdown electric field [61]. Therefore, the HfO_2_ layer is a promising candidate for a charge trapping layer. As mentioned above, the annealed LiNbO_3_ films exhibit high insulating properties and a ferroelectric phase, and they possess upward spontaneous polarization perpendicular to the substrate. Figure 15 shows the energy band diagram of the ferroelectric charge-trapping gate stacked LiNbO_3_/HfO_2_/Al_2_O_3_ films deposited on AlGaN/GaN layers, where E_g_ and χ were the bandgap energy and electron affinity, respectively [62,63,64]. Using the Fowler–Nordheim tunneling mechanism, the electrons residing within the 2DEG channel tunneled through the Al_2_O_3_ oxide layer and were trapped in the HfO_2_ charge-trapping layer. Furthermore, the trapped electrons were blocked by the LiNbO_3_ blocking layer to prevent their leakage to the gate metal. Consequently, the leakage current of the resulting devices could be significantly improved. As shown in Figure 15, polarized LiNbO_3_ not only assisted in depleting partial 2DEG but also possessed high insulating properties and formed a heterostructure with the HfO_2_ charge-trapping layer, so it could also serve as an excellent blocking layer. After that, two-finger gate windows were opened on the AlGaN layer under a SiO_2_ mask using a standard photolithography system, and the PEC etching method was employed to create 10 nm-deep two-finger gate-recessed regions. To fabricate the ferroelectric charge-trapping stack on the gate-recessed regions, an atomic layer system was used to deposit a 10 nm-thick Al_2_O_3_ tunnel oxide layer and a 4 nm-thick HfO_2_ charge-trapping layer in sequence. Then, a pulsed KrF laser deposition system was used to deposit a 40 nm-thick LiNbO_3_ blocking layer on the HfO_2_ layer. The sample was annealed in an oxygen ambient tube furnace at 600 °C for 30 min. Except that the above-mentioned fabrication processes of the ferroelectric charge-trapping stack were employed, the fabrication processes of the device shown in Figure 16 were the same as the GaN-based E-mode MOSHEMTs using the ferroelectric LiNbO_3_ layer only.

**Figure 17:**
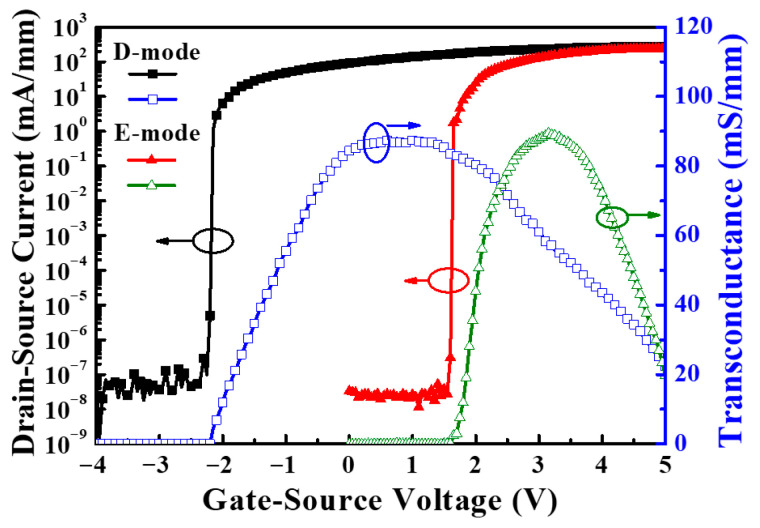
Drain-source current and extrinsic transconductance as a function of gate-source voltage of GaN-based MOSHEMTs with LiNbO_3_/HfO_2_/Al_2_O_3_ stack before and after initialization [57].

**Figure 18:**
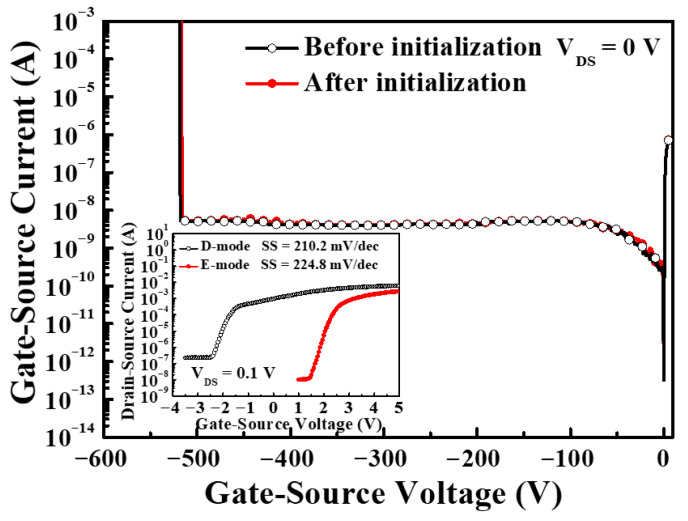
Gate-source current-gate-source voltage characteristics of GaN-based MOSHEMTs with LiNbO_3_/HfO_2_/Al_2_O_3_ stack before and after initialization [57].

**Figure 19:**
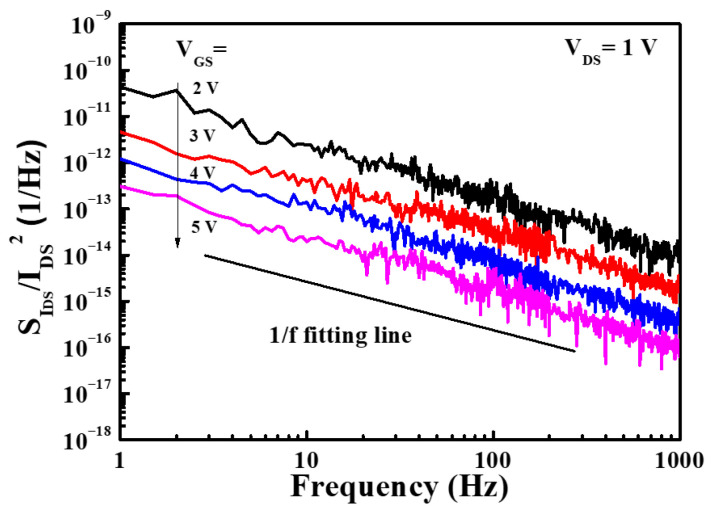
Normalized noise power density as a function of low frequency of GaN-based MOSHEMTs with LiNbO_3_/HfO_2_/Al_2_O_3_ stack [57].

Under the drain-source voltage of 7 V, Figure 17 shows the drain-source current and extrinsic transconductance as a function of gate-source voltage of the GaN-based MOSHEMTs with LiNbO_3_/HfO_2_/Al_2_O_3_ stack before and after initialization of gate-source voltage of 12 V for 10 ms. Before the device was initialized, the saturation drain-source current and the maximum extrinsic transconductance were 272.8 mA/mm and 87.4 mS/mm, respectively. The associated values of the initialized devices were 238.9 mA/mm and 89.5 mS/mm, respectively. The fact that the saturation drain-source current of the initialized devices was smaller than that before initialization was attributed to the inability to restore the original 2DEG concentration after initialization. Before and after the devices were initialized, the threshold voltage was −2.2 V and 1.6 V, respectively. The negative threshold voltage indicated that the D-mode behaviors resulted before the devices were initialized. When the devices were initialized, not only did their threshold voltage shift 3.8 V toward the positive voltage of 1.6 V, but enhancement characteristics were also achieved. Figure 18 shows the I_GS_ − V_GS_ characteristics of the GaN-based MOSHEMTs with LiNbO_3_/HfO_2_/Al_2_O_3_ stack operated at a V_DS_ of 0 V before and after initialization. It could be found that the devices had a high breakdown voltage of −520 V and a low gate-source leakage current of 5.1 nA before and after initialization. These similar experimental results demonstrated that the initialization process did not deteriorate the LiNbO_3_/HfO_2_/Al_2_O_3_ stack. Figure 19 shows the normalized noise power density ($S_{I_{DS}}(f)/{I_{DS}}^{2}$) as a function of frequency (f) of the devices operating at a drain-source voltage of 1 V and various gate-source voltages. Under the operation of V_DS_ = 1 V and V_GS_ = 5 V at a frequency of 10 Hz, the normalized power density was 8.6 × 10^−14^ Hz^−1^. The very low noise density demonstrated that the surface damage and interface states induced by creating the gate-recessed regions using the PEC etching method and the deposition of the LiNbO_3_/HfO_2_/Al_2_O_3_ stack could be significantly suppressed. In addition, the dominant noise originated from the flicker noise due to the quite well-defined tendency of 1/f with the normalized noise power density.

**Figure 20:**
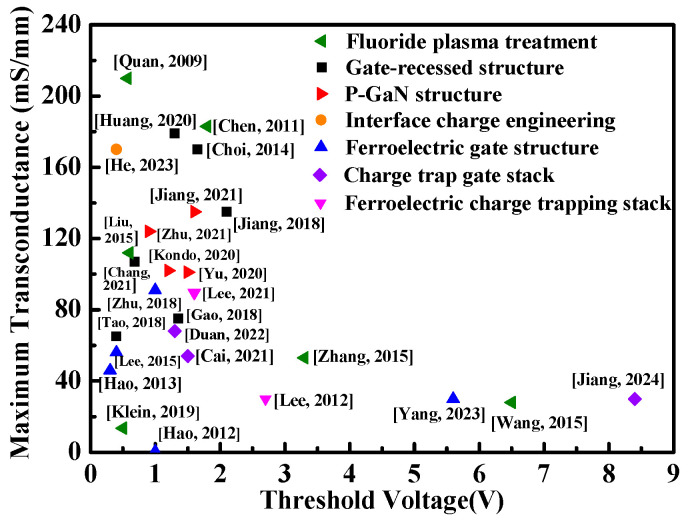
Performance comparison of GaN-based E-mode HEMTs fabricated using various methods. ◄: [34,43,70,71,72,80], ■: [65,66,67,68,79,81], ►: [73,74,75,76], ●: [78], ▲: [40,45,51,69,77], ♦: [44,56,58], and ▼: [57,59].

To compare the maximum transconductance and threshold voltage of the GaN-based E-mode HEMTs fabricated using various methods, the resulting performances are illustrated in Figure 20 [34,40,43,44,45,51,56,57,58,59,65,66,67,68,69,70,71,72,73,74,75,76,77,78,79,80,81].

## 5. GaN-Based Complementary MOSHEMTs and Monolithically Integrated Circuits

**Figure 21:**
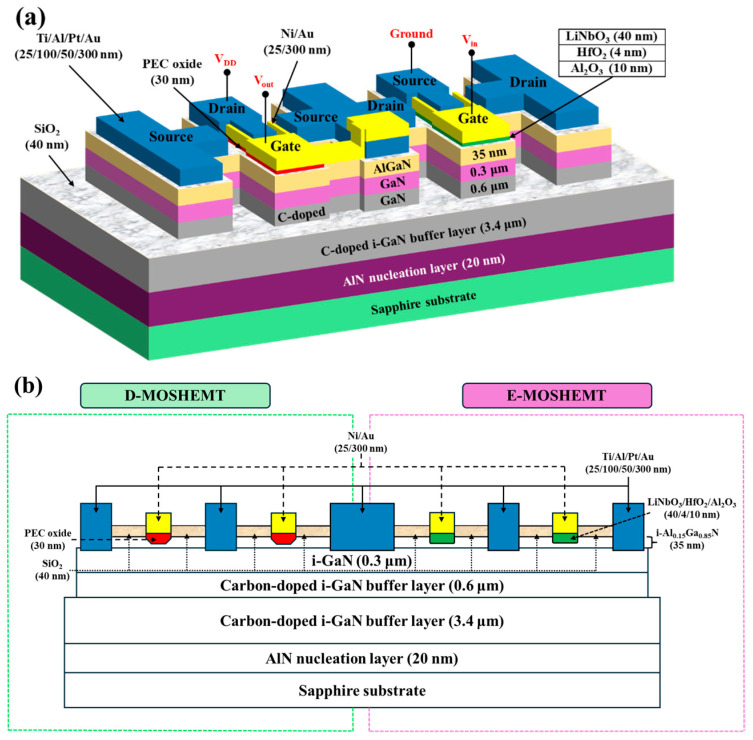
(**a**) Three-dimensional configuration and (**b**) cross-sectional configuration of GaN-based monolithic CMOS integrated circuit.

With continued scaling under Moore’s law, electronic device miniaturization has become mainstream and central to integrated circuit development. Recently, complementary metal-oxide-semiconductor (CMOS) architecture has served as the foundational building block of modern integrated circuits, such as power inverters, central processing units, memory arrays, and various sensors. Whereas traditional CMOS implementation in silicon-and III-V-based platforms integrate both n-channel and p-channel MOSFETs, GaN-based materials have emerged as critical semiconductors for MOSFETs, MOSHEMTs, and integrated circuits in low-noise, high-frequency, and high-power applications. However, to obtain high-performance GaN-based p-channel devices, achieving high hole concentration in GaN-based semiconductors via heavy p-type impurity doping remains difficult; moreover, their hole mobility is intrinsically low, and a two-dimensional hole gas has not yet been realized. Consequently, the fabrication of high-performance GaN-based p-channel MOSFETs, and thus fully GaN-based CMOS incorporating both n-channel and p-channel devices, remains an unmet challenge. To achieve a GaN-based CMOS circuit, the cascode configuration incorporating normally-on GaN-based HEMT and normally-off Si-based FET was reported previously [82]. However, this circuit exhibited drawbacks of package complexity, high parasitic inductance, and performance limitation by Si-based devices. To achieve GaN-based CMOS circuits, p-channel and n-channel MOSFETs were obtained by using a p-GaN layer and a Si-implanted p-GaN layer, respectively [83]. The low mobility properties of the p-GaN layer restricted the performance of the resulting circuits. To obtain the inherent advantages of a 2DEG channel, GaN-based complementary logic circuits were fabricated using the GaN-based E-mode HEMTs with a p-GaN gate as the n-FET and the GaN-based D-mode FET of an oxygen plasma-treated p-GaN layer as the p-FET [84]. Using the Si_3_N_4_ layer as the gate oxide layer and incorporating the fluoride-based treated E-mode and conventional D-mode of GaN-based MOSHEMTs, the GaN-based CMOS circuits with the 2DEG channel were demonstrated previously [85]. Using the LiNbO_3_ ferroelectric gate to fabricate E-mode MOSHEMTs and incorporating D-mode MOSHEMTs with a PEC-oxidized gate oxide layer, the GaN-based CMOS circuit with the 2DEG channel was also reported previously [86]. In addition, using Al_2_O_3_ as the gate oxide layers and using shallow and deep gate-recessed regions to, respectively, fabricate D-mode and E-mode MOSHEMTs, the GaN-based CMOS circuits were realized [82]. Recently, based on the inherent properties of the 2DEG channel, GaN-based CMOS HEMTs were successfully fabricated and reported by incorporating the D-mode MOSHEMTs manufactured using the PEC oxidation method and the E-mode MOSHEMT manufactured using the PEC etching method and LiNbO_3_/HfO_2_/Al_2_O_3_ stack [87]. Figure 21a,b show the three-dimensional configuration and cross-sectional configuration of the GaN-base monolithic CMOS-HEMTs integrated circuits, respectively.

**Figure 22:**
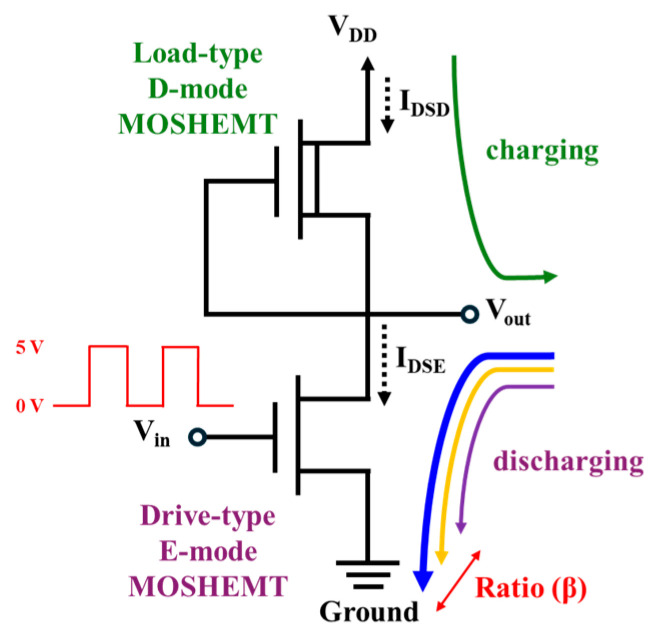
Common-source inverter circuit using GaN-based CMOS-HEMTs.

**Figure 23:**
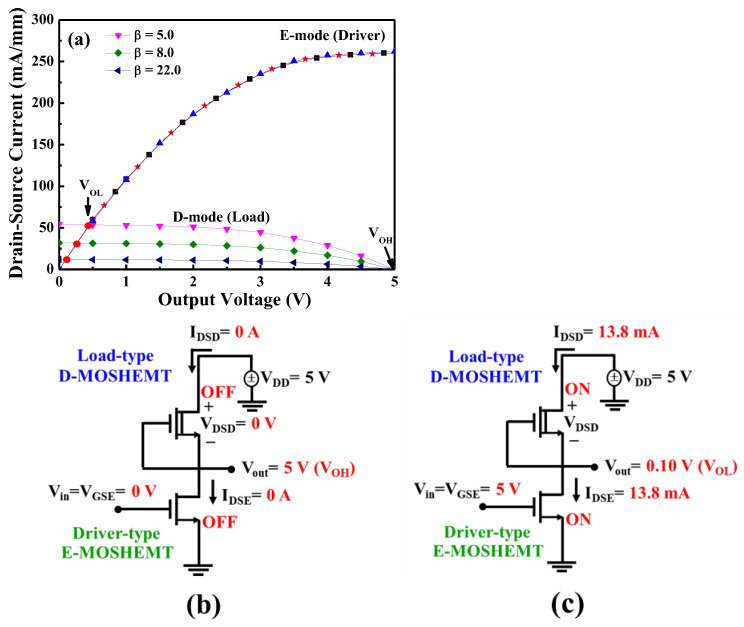
(**a**) Load line characteristics of inverter with various β values. The marks (■, ★, and ▲) indicated I_DSE_ for β value of 5.0, 8.0, and 22.0, respectively. and schematic circuit configuration of (**b**) V_in_ = 0 V and (**c**) V_in_ = 5 V [87].

**Figure 24:**
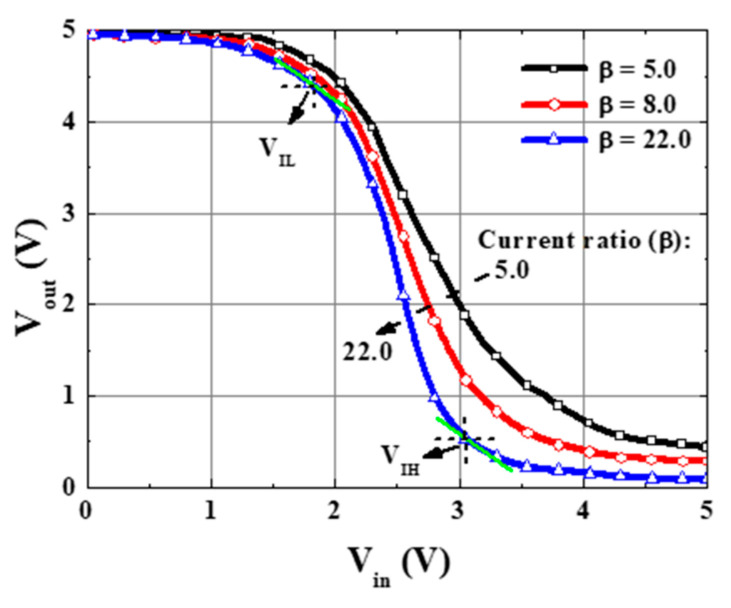
Static voltage transfer characteristics of inverter with various β values [87].

Figure 22 illustrates the common-source inverter circuit constructed by the GaN-base monolithic CMOS-HEMTs, which integrated the D-mode MOSHEMT as the load and the E-mode MOSHEMT as the input driver. The gate electrode was shorted with the source electrode of the D-mode MOSHEMTs and connected with the drain electrode of the E-mode MOSHEMTs using Ni/Au metals. To achieve current matching between them through precise tuning of the remaining AlGaN barrier thickness in the D-mode device via the PEC etching process, an inverter using the GaN-based CMOS-HEMTs without the need for p-channel GaN devices was thereby realized. The drain-source current of the load D-mode MOSHEMTs could be controlled by adjusting the remaining AlGaN barrier thickness instead of the conventional method by changing the gate width. Consequently, not only could the drain-source current ratio between the D-mode and E-mode MOSHEMTs be controlled as needed, but also D-mode and E-mode devices could have the same gate width to minimize the dimension of the GaN-based CMOS circuits. For the inverter shown in Figure 22, when the V_DD_ was 5 V and the V_in_ was the pulsed voltage with a magnitude of 5 V, the load characteristics of the drain-source current as a function of output voltage V_out_ are shown in Figure 23 [87], where V_out_ was the drain-source voltage V_DSE_ of the E-mode device and equaled to V_DD_ − V_DSD_, and where V_DSD_ was the drain-source voltage of the D-mode device. Under the drain-source voltage V_DSD_ of 5 V and the gate-source voltage V_GSD_ of 0 V, the saturation drain-source current I_DSD_ was 11.8, 32.5, and 52.6 mA/mm for the GaN-based D-mode MOSHEMTs with the remaining AlGaN thickness of approximately 8.0, 10.0, and 12.0 nm, respectively. It could be found that the saturation drain-source current not only decreased with decreasing the remaining AlGaN thickness, but also was controlled by the PEC etching method. Under the operation of the drain-source voltage V_DSE_ of 5 V and the gate-source voltage V_GSE_ of 5 V, the drain-source current I_DSE_ of the GaN-based E-mode MOSHEMT was 260.5 mA/mm. By defining β as the ratio of I_DSE_/I_DSD_, the β values were 5.0, 8.0, and 22.0 corresponding to the drain-source current of the D-mode devices with the remaining AlGaN thicknesses of 12.0, 10.0, and 8.0 nm, respectively. Under the operation of V_in_ = V_GSE_ = 0 V, because the E-mode and D-mode devices operated in cutoff mode, the I_DSE_ and I_DSD_ were 0 A, and the V_DSD_ was 0 V. Therefore, the V_out_ was located at a high output voltage V_OH_ and equaled the V_DD_ = 5 V. These operations are illustrated in Figure 23b. On the other hand, when the Vin and V_DD_ were 5 V, the E-mode device operated in saturation mode, and the I_DSD_ equaled I_DSE_. Consequently, the quiescent point operated at the intersection point of the load line characteristics. As shown in Figure 23, the low output voltage V_OL_ decreased with an increase in β value. The operation of the inverter with a β value of 22.0 is shown in Figure 23c. As shown in Figure 23a, when the V_in_ was 5 V, the V_out_ of the inverter with β values of 5.0, 8.0, and 22.0 had the I_DSD_ = I_DSE_ of 53.2 mA, 32.8 mA, and 13.8 mA, and V_out_ of 0.45 V, 0.28 V, and 0.10 V, respectively. It was found that the V_out_ was not 0 V when the V_in_ was 5 V. Consequently, they had the disadvantage of static power loss of I_DSE_ = V_out_. In the inverter, the static power loss decreased with an increase in β value. Furthermore, the output swing voltage (V_DD_ − V_OL_) increased by increasing the β value. Figure 24 shows the static voltage transfer characteristics of the inverter with various β values under the operation of V_DD_ = 5 V. In Figure 24, the high input voltage (V_IH_) and the low input voltage (V_IL_) were defined as the voltage corresponding to the operating point with a slope of −1. In addition, the low output voltage (V_OL_) and the high output voltage (V_OH_) were defined as the output voltage corresponding to the input voltage of 5 V and the input voltage of 0 V, respectively. When the output voltage V_out_ of the inverter with various β values was 2.5 V, Table 1 lists the associated output swing voltage (V_DD_ − V_OL_), high noise margin voltage (NM_H_ = V_OH_ − V_IH_), low noise margin voltage (NM_L_ = V_IL_ − V_OL_), and Vin values. By designing the inverter with the β value of 22.0, the V_in_ was very close to the V_out_ = V_DD_/2. It was demonstrated that the unskewed inverter could be implemented by matching the associated current of the D-mode and E-mode MOSHEMTs to achieve the optimal β value.

## 6. Conclusions

In this work, the PEC etching method and the PEC oxidation method were developed for fabricating GaN-based MOSHEMTs. PEC etching was utilized to create gate-recessed regions, and the PEC oxidation method was utilized to directly grow the gate oxide layer. Due to the minimization of surface damage and interface state density, the resulting high-performance GaN-based D-mode, E-mode, and complementary MOSHEMTs were manufactured and demonstrated. To manufacture GaN-based D-mode MOSHEMTs, if the thin AlGaN barrier layer was grown, the PEC oxidation method could be employed to directly grow the gate oxide layers and simultaneously create gate-recessed regions. However, if the thick AlGaN barrier layer was grown in the epitaxial layers, the PEC etching method could first be used to etch the partial AlGaN barrier layer. Then, the PEC oxidation method could be used to simultaneously create gate-recessed regions and directly grow the gate oxide layer. To fabricate GaN-based E-mode MOSHEMTs, owing to the high breakdown insulator and the direction of the high polarization opposite to the polarization of the AlGaN/GaN heterostructure, the annealed LiNbO_3_ layer was utilized as the ferroelectric gate layer and the gate oxide layer. To further improve the performance of GaN-based E-mode MOSHEMTs, the ferroelectric charge-trapping gate stack was developed and demonstrated. To take the inherent advantages of the LiNbO_3_ layer in the GaN-based E-mode MOSHEMTs, the LiNbO_3_ film was deposited as the blocking layer on the charge-trapping gate stack that used the Al_2_O_3_ film as the tunneling layer and the HfO_2_ film as the charge trapping layer. Consequently, the electrons residing in the 2DEG channel could tunnel through the Al_2_O_3_ tunnel layer and were trapped in the HfO_2_ charge-trapping layer. The ferroelectric LiNbO_3_ blocking layer not only assisted in depleting some of the electrons residing in the 2DEG channel, but its high insulating properties and high heterogeneity with the HfO_2_ charge-trapping layer could also effectively prevent the trapped electrons from leaking to the gate electrode. Therefore, the gate leakage current could be significantly reduced. When high-performance GaN-based D-mode and E-mode MOSHEMTs manufactured using the PEC-etching method and the PEC-oxidation method were integrated to form GaN-based CMOS HEMTs, they also exhibited high-performance function when applied to an inverter. Based on the advantages of flexible application, low damage, and low defects, it is seen that using the PEC etching method to create the gate-recessed regions can avoid surface damage to GaN-based layers, and the oxide layer directly grown using the PEC oxidation method can be used not only as the gate oxide layer but also as the surface passivation layer of the resulting devices. In this work, we only reported that we applied the PEC oxidation method and the PEC etching method to our fabricated GaN-based D-mode, E-mode, and CMOS HEMTs and verified their functionality and characteristics. However, given the excellent capabilities demonstrated by the PEC etching method and the PEC oxidation method in GaN-based device manufacturing, it is expected that they will also be useful in the fabrication of other devices.

## Figures and Tables

**Table 1:** Performances of inverters with various β values.

Current Ratio β	V_OL_ (V)	Output Swing (V)	NM_H_ (V)	NM_L_ (V)	V_in_ as V_out_ = V_DD_/2 (V)
5.0	0.45	4.55	1.44	1.48	2.80
8.0	0.28	4.72	1.80	1.62	2.60
22.0	0.10	4.90	1.99	1.73	2.49

## Data Availability

The data presented in this study are available on request from the corresponding author.
